# The Bedford Alum and Iron Spring

**Published:** 1879-04-20

**Authors:** J. J. Moorman

**Affiliations:** Vircinia


### *THE BEDFORD ALUM AND IRON SPRING.*

BY PROF. J. J. MOORMAN, M.D., OF VIRGINIA.

The aluminous sulphated chalybeate waters have for half a century occupied a conspicuous place among the valuable medicinal waters of our highly-favored mineral-water country. The Bedford Alum and Iron Springs, belonging as it distinctly does, to this class of waters, and showing an analysis not inferior to the best of its class, may well, on account of its constituent ingredients, stand in fair competition with similar waters whose virtues have been long known and appreciated by the invalid public, and therefore might confidently challenge a large public confidence in their efficacy. While such is a fair theory in reference to the efficacy of this water, actual experience of its use for near thirty years has fully verified the hopes that its analysis created. Both the analysis, then, and practical use of this water, go to prove its great efficacy in a wide circle of disease, and to one disease especially, which, without the use of this class of waters, would stand, as it has hitherto stood, the *opprobria medicorum* of the healing art. I allude to scrofula, in its well-developed form. For this affection, waters of this class are altogether the best remedies known to the profession; and if there were no other diseases for which such waters are remedial, their efficacy in this would give them high distinction. But their remedial power is by no means confined to this formidable malady. It reaches and overcomes many other diseases and morbid states of the system.

The immediate therapeutic effects of this class of waters as I have remarked, in my volume on the mineral waters of the United States and Canada, are those of a *febrifuge tonic*, resembling the action of some of our best vegetable medicines of this class, and especially of quinine, but superior to them in some respects from their speeific tendency to the bowels and kidneys. By their diffusing, astringent and tonic powers, they resolve congestions of engorged *viscera*, and remove sub-acute inflammation, thus relieving and giving activity to the fluids they fill up the superficial capillaries and veins, and give a full, slow pulse, with warm surface and soft skin. They are not purgative waters, except in increased quantity; but sometimes gently open the bowels when taken in smaller doses. Their action upon the kidneys is generally prompt, their action upon the skin is secundary; and when it occurs, is the result of their sanitary action upon the blood vessels by resolving inflammations and congestions, and hencc such action may always be regarded as favorable indications in the case.

These waters are advantageously prescribed in many chronic affections of the skin and glandular system. Even *lupus* and other malignant ulcerations are cured by them. In chronic diarrhœa they are often administered to advantage, and the same may be said of chronic irritations of the kidneys, bladder and urethra. In paucity and poverty of the blood; in the chlorotic condition of young females, and in several forms of uterine derangement, as well as in anæmic conditions, with loss of tone in the nervous system, they may be prescribed with great advantage. The salts, or mass obtained by evaporation of the water, may be re-dissolved and used to advantage when the water is not at hand. For many years I have been in the habit of using with great advantage a strong solution of such salts as a local application to indolent ulcerations of the skin, and especially to those of a scabeous character. To the scaly and irritable eruptions that not unfrequently appear upon the cheeks, and sometimes the nose of elderly persons, giving uneasiness lest they should prove malignant, I have employed frequently a strong solution of such salts with the most satisfactory results.

In various chronic affections of the digestive organs under the generic name of dyspepsia, either simple or implicating other organs, and especially that form of such depravities known as *gastralgia*, or *nervous dyspepsia* such waters constitute a valuable remedy. The same may be said of them in *mesenteric* affections, and particularly in persons old or young, of scorbutic tendencies.—*Maryland Med. Journal.*

---

**Pilocarpin as an Abortifacient.**—A late number of the Allgemein Med. Central Zeitung quotes four cases recently reported by various observers, where the hypodermic use of pilocarpin in pregnant women led to abortion. One case was four months gone, and aborted after the ninth injection. This should be an important caution against using this agent, which is growing in popularity, in pregnant women. As to whether it will prove of service in cases where the production of abortion is indicated for legitimate reasons, no observations have been reported.—*Med. Rep.*

